# Super-refractory status epilepticus, rhabdomyolysis, central hyperthermia and cardiomyopathy attributable to spinal anesthesia: a case report and review of literature

**DOI:** 10.1186/s12871-024-02485-x

**Published:** 2024-04-06

**Authors:** N. D.B. Ehelepola, R. M.D.C. Ranathunga, A. B. Abeysundara, H. M.R.P. Jayawardana, P. S.K. Nanayakkara

**Affiliations:** 1 Teaching (General) Hospital – Peradeniya, Peradeniya, Sri Lanka; 2 Faculty of Medicine, University of Peradeniya, Peradeniya, Sri Lanka

**Keywords:** Bupivacaine, Spinal anesthesia, Status epilepticus, Rhabdomyolysis, Hyperthermia, Idiosyncrasy, Cardiomyopathy, Side effect, Case report

## Abstract

**Background:**

There are only six past reports of super-refractory status epilepticus induced by spinal anesthesia. None of those patients have died. Only < 15 mg of bupivacaine was administered to all six of them and to our case. Pathophysiology ensuing such cases remains unclear.

**Case presentation:**

A 27 year old gravida 2, para 1, mother at 37 weeks of gestation came to the operating theater for an elective cesarean section. She had no significant medical history other than controlled hypothyroidism and one episode of food allergy. Her current pregnancy was uneventful. Her American Society of Anesthesiologists (ASA) grade was 2. She underwent spinal anesthesia and adequate anesthesia was achieved. After 5–7 min she developed a progressive myoclonus. After delivery of a healthy baby, she developed generalized tonic clonic seizures that continued despite the induction of general anesthesia. She had rhabdomyolysis, one brief cardiac arrest and resuscitation, followed by stress cardiomyopathy and central hyperthermia. She died on day four. There were no significant macroscopic or histopathological changes in her brain that explain her super refractory status epilepticus. Heavy bupivacaine samples of the same batch used for this patient were analyzed by two specialized laboratories. National Medicines Quality Assurance Laboratory of Sri Lanka reported that samples failed to confirm United States Pharmacopeia (USP) dextrose specifications and passed other tests. Subsequently, Therapeutic Goods Administration of Australia reported that the drug passed all standard USP quality tests applied to it. Nonetheless, they have detected an unidentified impurity in the medicine.

**Conclusions:**

After reviewing relevant literature, we believe that direct neurotoxicity by bupivacaine is the most probable cause of super-refractory status epilepticus. Super-refractory status epilepticus would have led to her other complications and death. We discuss probable patient factors that would have made her susceptible to neurotoxicity. The impurity in the drug detected by one laboratory also would have contributed to her status epilepticus. We propose several possible mechanisms that would have led to status epilepticus and her death. We discuss the factors that shall guide investigators on future such cases. We suggest ways to minimize similar future incidents. This is an idiosyncratic reaction as well.

**Graphical Abstract:**

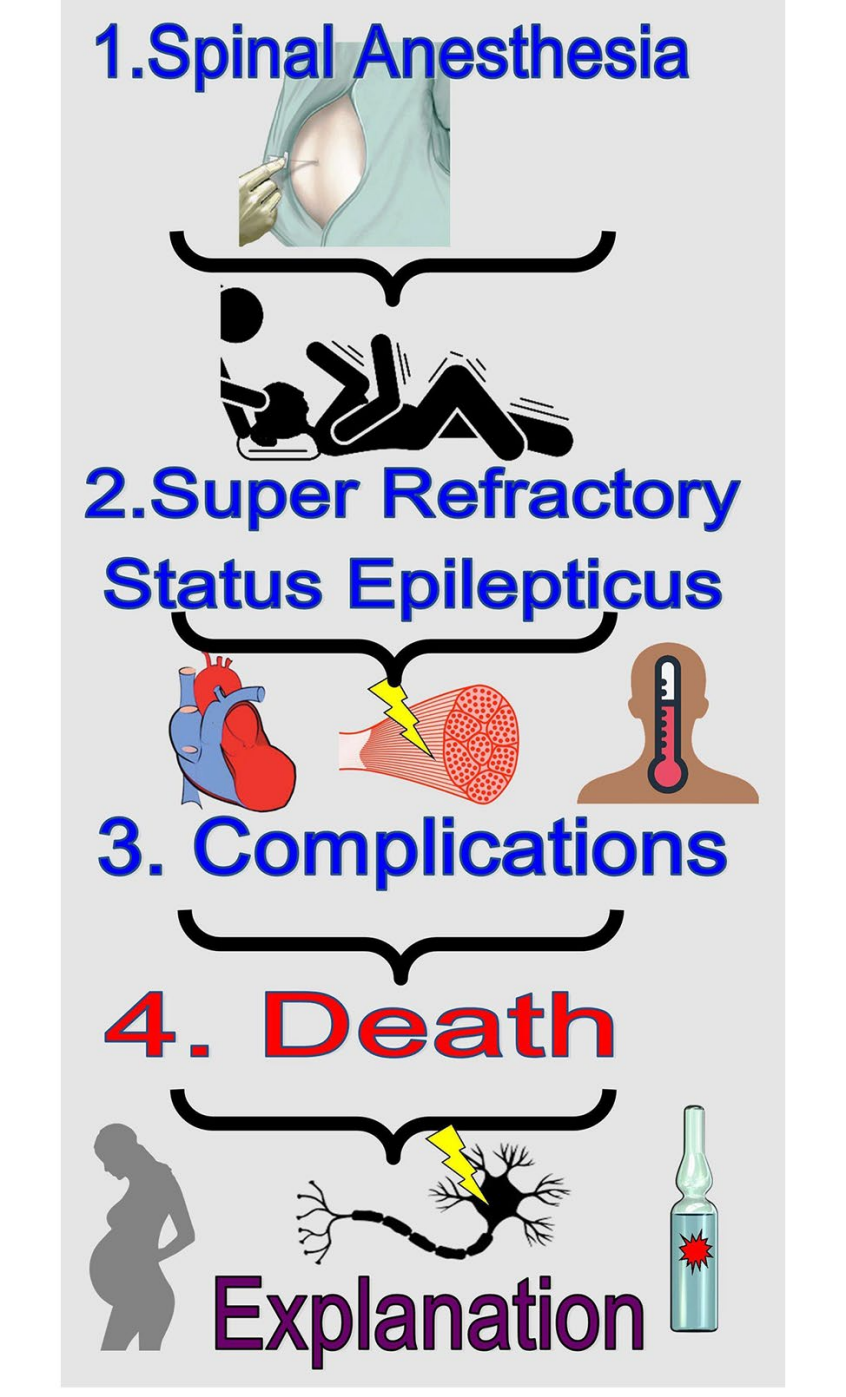

**Supplementary Information:**

The online version contains supplementary material available at 10.1186/s12871-024-02485-x.

## Background

There are only a few past reports on status epilepticus attributed to drugs used for spinal anesthesia [1–7]. Out of those, six past reports are of super-refractory status epilepticus cases attributed to bupivacaine and those patients have recovered [1–6]. Pathophysiology resulting in such cases notwithstanding only < 15 mg of bupivacaine administered, remains unclear. A status epilepticus that continues for ≥ 24 h despite anesthetic treatment, or recurs on an attempted wean of the anesthetic drugs is defined as super-refractory status epilepticus [5, 8]. There are no past reports of the combination of super-refractory status epilepticus, rhabdomyolysis, central hyperthermia and cardiomyopathy resulting in the death of a previously healthy person attributed to a spinal anesthetic agent. This death and a few other subsequent deaths due to drug side effects and the alleged poor quality of the responsible drugs generated a huge outcry in most national news media. Many social media sensationalized the issue and it was debated in Sri Lanka’s parliament as well. That resulted in anxiety among patients undergoing anesthesia, anesthesiology doctors and people utilizing public hospitals. There were some public protests in Sri Lanka in 2023 regarding this issue. This crisis was in the news in some other countries as well [9].

## Case presentation

A 27 year old pregnant woman, height 151 cm, weight 78 kg (Body Mass Index 34.2), in 37 weeks of gestation, came to the operating theater for an elective cesarean section (EL/LSCS) after routine preparation. She is the mother of a five year old child and this is her second pregnancy. Other than hypothyroidism controlled with thyroxin for the past three months she had no significant medical history or history of substance abuse. However, after the below mentioned incident her father told doctors that she had one allergic reaction to pineapple during her childhood which did not require hospital care. Therefore, her ASA grade was 2. She underwent spinal anesthesia in the sitting position, lumbar 4–5 level. A 25G pencil point needle was used. After verification, 2.5 cc of 0.5% heavy bupivacaine = 12.5 mg of bupivacaine (ZUPIVAC H, batch No.DP2203) without adjuvants was administered. Adequate analgesia was achieved up to thoracic dermatome7 level and surgery was started. After 5–7 min, she complained of a backache and a “discomfort” which she could not explain. Then she developed occasional myoclonus in her upper limbs and upper trunk and that once involved legs as well. Myoclonus was momentarily controlled by 2 mg of intravenous (IV) midazolam. Myoclonus progressed in magnitude and frequency. When asked, she denied any circumoral numbness and power of the grip of her both hands was normal. A crying baby was delivered 10 min after anesthesia. Oxytocin was administered and the mother could verbally communicate with the medical officer-anesthesiology. Then her eyes deviated upwards, she could not respond verbally and developed generalized tonic clonic seizures (GTCS). General anesthesia was inducted using thiopentone, she was intubated with suxamethonium and ventilated. Her blood pressure remained 130/80 mmHg − 140/90 mmHg, heart rate in 110–140/min range, ECG(EKG) showed sinus tachycardia, and her peripheries were warm and flushed. ETCO_2_ was 55–65 mmHg. Her both pupils were 3 mm and sluggishly reacting to light. Her random blood sugar level was 134 mg/dl. As she developed some bilateral rhonchi (lungs), intravenous (IV) hydrocortisone, chlorpheniramine was administered and she was nebulized with ipratropium. There was uterine atony and bleeding. IV oxyticin, intramuscular ergometrin and per rectal misoprostol were administered and one unit of blood was transfused. Two and a half hours after anesthesia she was taken to the ICU and electively ventilated. Her arterial blood pH was 7.03 and her lactate level was 9.6 mmol/l. Acidosis was corrected with IV sodium bicarbonate. Her chest X ray (CXR) showed a globular heart and haziness in the left lung field, NCCT did not show any abnormality in the brain, protein was +++ in urine; and there was no proteinuria before surgery (many public hospital laboratories of Sri Lanka report proteinuria in a scale from trace to +++. Three + means very high levels). She developed hypotension and norepinephrine infusion was started at 0.3 µg/kg/min rate. She was assessed by consultants/professors in anesthesiology, obstetrics, internal medicine and neurology and by other doctors on the same day. The differential diagnoses of the multi-disciplinary team were an adverse reaction to bupivacaine, amniotic fluid embolism, normotensive eclampsia and previously undiagnosed brain pathology. Our primary goal was to control her seizures while providing life support therapy. She was administered thiopentone, midazolam, MgSO4, levetiracetam, morphine, mannitol and antibiotics etc. Approximately once in four hours thiopentone infusion was withheld to assess her for seizures and to avoid myocardial depression. Atracurium was administered as 25 mg boluses. Whenever the effect of atracurium was weaning she had continuous GTCS on days 1–3 sometimes even while thiopentone was being infused. 20% fat emulsion was not available at our and nearby hospitals at that period of time. Her ETCO_2_ remained in 34 -40mmHg range after admission to the ICU.

On the following morning (day 2) she had an episode of bradycardia followed by cardiac arrest and was successfully resuscitated within five minutes. Epinephrine and dopamine infusions were added after this. A 2D echocardiogram performed by one of the ICU doctors did not show hypokinesia in the ventricular walls. Her tachycardia persisted and she developed fever spikes. ST elevations appeared in lead 1, aVL of the ECG(EKG). Therefore, aspirin, clopidogrel and atorvastatin were added. Status epilepticus continued thus levetiracetam dose was increased, IV phenytoin and NG lacosamide were added.

On day3,the seizure frequency decreased. Repeated NCCT did not show any hemorrhages or infarcts in the brain. ST elevations were seen in leads I, aVL, V5-V6 in ECG and troponin I was > 50 ng/ml. Hence, cardiologists opinion was obtained. Creatine kinase (CK) level was 21,420 U/L and alkaline diuresis was started. She had two high fever spikes per day, each > 40^0^C on day 2 and day3.

On day 4, she was seizure free but had hyperpyrexia (once 42.8^0^C) despite active cooling. Lumbar puncture was done and CSF analysis did not show evidence of infection. Same morning she suffered a cardiac arrest, resuscitation was continued for more than an hour but was unsuccessful.

After the drop in day 2, her blood pressure was maintained with three inotropes infused at the following rates. Norepinephrine at 0.3–0.6 µg/kg/min, epinephrine at 0.3–0.6 µg/kg/min and dopamine at 20 − 10 µg/kg/min. Her SpO_2_ remained > 92%, her urine output was 40-100 ml/hour and serum creatinine levels were 87–123 micromoles/l during her ICU stay. Her day 1 prothrombin time was 19.2 s and INR was 1.62. Thiopentone would have contributed to this prolongation of INR [10]. INR was ≤ 1.5 after day 2. Her activated partial thromboplastin time was 26.5 s on day1 and remained normal. EEG and MRI brain could not be done before her death.

The autopsy was performed by a consultant judicial medical officer. Both lungs were congested and exuded frothy fluid on sectioning and the liver was enlarged and soft. No other remarkable abnormalities were detected including in the brain and heart. However, spinal cord was not dissected.

Cerebrum and cerebellum histopathology were unremarkable. Widespread early ischemic changes were seen in the myocardium. Centrilobular necrosis with occasional bridging necrosis in 20% of the liver and mild acute tubular necrosis in the kidneys were seen. Extensive degenerative changes were seen in skeletal muscles. Pulmonary edema, evidence of mild pneumonia and focal hemorrhages were seen in the lungs. Multiple bone marrow emboli were seen in the lungs and heart, consistent with changes associated with prolonged CPR. All changes seen were acute changes.

Samples from all three batches of ZUPIVAC H at our hospital were analyzed by the National Medicines Quality Assurance Laboratory (NMQAL) of Sri Lanka. Samples confirmed USP bupivacaine and other specifications but batch No.s DP 2203 and DP 2202 failed to confirm dextrose specifications. Thereafter, samples were re-analyzed at the Therapeutic Goods Administration (TGA) laboratories of the Australian government. When batch No. DP 2203 was tested according to the relevant USP monograph to validate the medicine and its ingredients, the medicine passed the tests applied to it (it passed the quality test). However, there is no impurity test in the USP monograph. Considering the very unusual and lethal nature of this adverse drug reaction we have requested TGA-Australia look for any impurities as well. Fulfilling our request, they have identified an impurity that is suspected to be structurally related to bupivacaine in batch No. DP 2203. It’s content was estimated to be 0.9% relative to bupivacaine. They have confirmed that this impurity is not mepivacaine. This impurity was not specifically identified in any of the pharmacopoeias available at TGA-Australia. Despite further testing, they were unable to identify what exactly this impurity is. TGA-Australia has noticed another unusual thing. That is although the ZUPIVAC H label claims compliance with the USP for the finished product, they claim compliance with the Indian Pharmacopoeia (IP) for raw materials. The full report is given as a supplementary file.

## Discussion and conclusions

Our discussion has eight subheadings. Those are; key facts of the case report, how did we conducted a systematic literature review to find similar reported cases, the relationship of the presented case with the existing literature, circumstantial evidence for and against suspecting heavy bupivacaine (ZUPIVAC H) as the cause of her seizures, explanation of the patient’s clinical and autopsy findings, differential diagnoses we considered at different stages, discussion of central nervous system toxicity by bupivacaine and possible mechanisms of toxicity and lessons to be learned and the way forward.

### Key facts of the case report

An ASA grade 2 pregnant mother underwent a routine a spinal anesthesia for a cesarean section and adequate anesthesia was achieved. After 5–7 min, she developed a progressive myoclonus that evolved into generalized tonic clonic seizures after delivery. Her seizures continued despite administering general anesthesia plus multiple anticonvulsants. She had rhabdomyolysis, one brief cardiac arrest, stress cardiomyopathy, central hyperthermia and died on day four. There were no significant radiological, macroscopic or histopathological changes in her brain or biochemical changes that explained her super refractory status epilepticus. Heavy bupivacaine samples from the same batch used for this patient were analyzed by two specialized laboratories. One laboratory reported that samples failed to confirm USP dextrose specifications. Bupivacaine samples passed all standard USP quality tests at the other laboratory. Nevertheless, they managed to detect an unidentified impurity in the medicine.

### How did we conducted a systematic literature review to find similar reported cases

Super-refractory status epilepticus is rare and many doctors we know do not use that term. Thus, we decided to search for the word “status epilepticus” and select super-refractory status epilepticus cases out of those. We conducted a literature survey in several online databases in February 2024 for the keywords “status epilepticus”,“spinal anesthesia” and “case report” combined using the Boolean operator “AND”. Those databases were Google Scholar, PubMed, Europe PubMed Central, CNKI, ScienceDirect and DOAJ. A total of 576 articles were identified. Abstracts of each were read and 564 irrelevant articles were excluded. We used the PICO (P: patient/population/problem, I: intervention, C: comparison/control—O: outcome) framework to determine inclusion criteria [11]. Accordingly, we selected patients undergoing spinal anesthesia as our population, spinal anesthesia as intervention and super-refractory status epilepticus cases as the outcome. Out of the remaining 12, duplicates were removed, full articles were read and five cases that fulfill the definition of super-refractory status epilepticus attributed to a spinal anesthesia drug were identified. They were our references 2,3,5 and 6. The snowballing of those led to identification of our reference1. First and third authors independently did this process and obtained the same results.

**Fig. 1:**
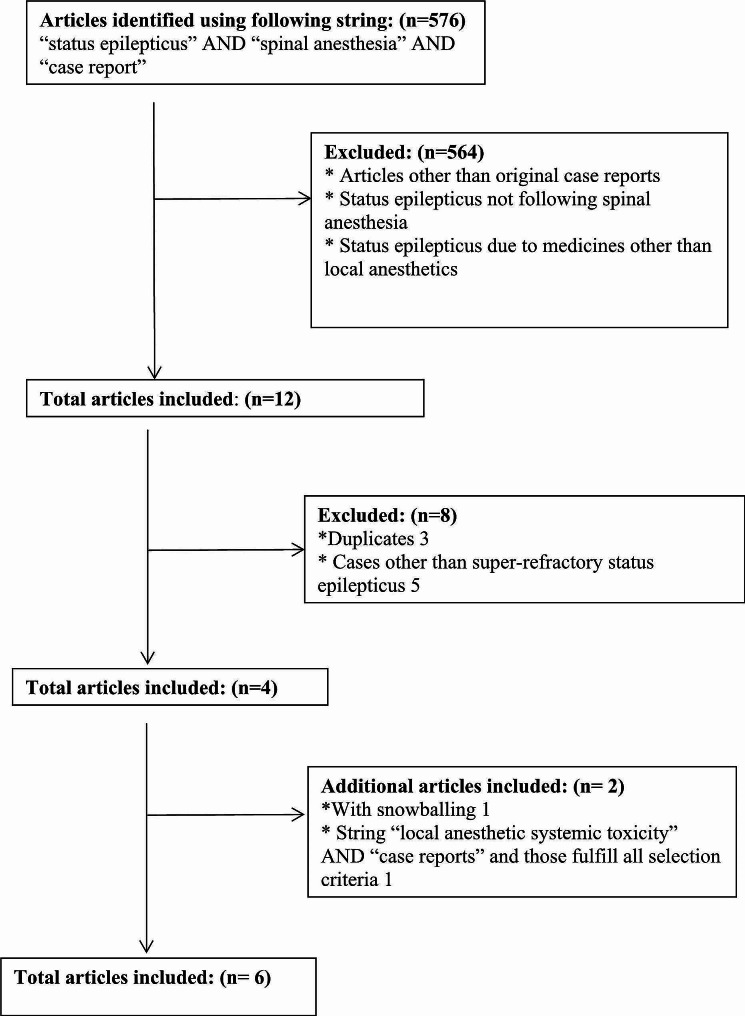
The flowchart of systematic literature review process

The flowchart of the process is shown in Fig. 1.

In cases of reference1 and 6 the duration of seizures was not clearly mentioned, but their authors state that seizures continued despite the administering of anesthetic drugs. Super-refractory status epilepticus is primarily defined by failure to respond to appropriate treatment, not by seizure duration [12]. Hence, we included those two cases. The case of reference 7 has seizures for a few hours; hence we did not include that. Nonetheless, it is the first report (1994) of a status epilepticus (refractory status epilepticus) case following spinal anesthesia we found [7]. That patient had a history of seizures and tertracaine was used for anesthesia.

Earlier, we did a nonsystematic literature survey employing various strings. Among results for the string “local anesthetic systemic toxicity” AND “case reports” we have identified our reference 4. Key findings from past six reports of super-refractory status epilepticus attributable to spinal anesthesia are summarized in Table 1. The authors of some of those papers do not classify their cases as super-refractory status epilepticus cases.

**Table 1:** Key findings in past reports of super-refractory status epilepticus attributable to spinal anesthesia

Year and Country	Drugs administered	Clinical features	Investigations	Medical History
2014 Turkey [1]	Heavy bupivacaine 12.5 mg (Marcaine)	Perineal pain, myclonus of leg followed by status epilepticus	The contrast MRI-Brain, MRI venography and arteriography were normal. EEG showed a slow wave activity originating from the bilateral frontal areas. CSF analysis was normal. CK was > 4000U/l.	Nothing
2020 India [2]	heavy bupivacaine 11 mg	LSCS-After delivery of the baby mother had headache and neck rigidity followed by status epilepticus	MRI brain:leptomeningitis with associated evolving cerebritis. CSF analysis was normal	Nothing
2022 Belgium [3]	heavy bupivacaine 15 mg with 0.25 µg sufentanil	myoclonus leading to status epilepticus	CT and MRI of the brain were normal. CSF analysis was normal. EEG:epileptic activity	hysterectomy and supraventricular tachycardia
2022 Portugal [4]	heavy bupivacaine 11.25 mg and 3.75 µg sufentanil	Tremor > converted to general anesthesia > status epilepticus. Also had cardiac effects including ventricular fibrillation	CT and MRI of the brain: hypoxic-ischemic brain injury	osteoarthritis
2022 India [5]	heavy bupivacaine 10 mg	Perineal discomfort > up rolling of eyes > rigidity > GTCS > anterograde amnesia	CSF analysis was normal. MRI: lesions suggestive of vasogenic edema with a distribution typical of posterior reversible encephalopathy syndrome. EEG(day 11) normal	preeclampsia and (on labetalol)
2022 India [6]	heavy bupivacaine 11 mg	itching in the coccyx region with flushing of face > restless > GTCS	Not mentioned	Nothing

### Relationship of the presented case with the existing literature

Similar to our case, all six cases were anesthetized using heavy bupivacaine. Only < 15 mg of bupivacaine was administered to all of them. However, sufentanil was added to heavy bupivacaine in two cases unlike us. None of them or our patient had any history of past seizures. Interestingly, 5/6 patients were women. Three past cases like our patient, had seizures minutes after spinal anesthesia while undergoing cesarean sections. One of them had a history of preeclampsia unlike our case. 3/6 had discomfort in the perineum or coccyx region before seizures and our patient complained of a discomfort that she could not describe. Like in our case, 3/6 had myoclonus or tremors prior to seizures. All past cases survived. None of the past cases had the combination of rhabdomyolysis, a brief cardiac arrest followed by stress cardiomyopathy and central hyperthermia.

There are several other reports of seizures associated with spinal anesthesia [3, 13–15]. Only two cases of seizures following spinal anesthesia was reported between 1945 and 1962 according to one author [13]. Reports have become more frequent lately (Table 1). A recent paper summarized 30 publications describing 31 patients and two cats who had had GTCS or myoclonus following spinal anesthesia [15]. Out of those cases, 64% resolved spontaneously and promptly and in 71% of cases neurotoxicity manifested 30 min after spinal anesthesia, in contrast to our case [15]. In the literature, there is a spectrum of cases varying from mild self-limiting myoclonus probably originating at the spinal cord level, to super-refractory status epilepticus cases like our case. We think the likely explanation for our case as follows. Initially, she would have had spinal myoclonus which was momentarily controlled by Midazolam. Although its density was higher than that of the CSF (as explained later), a minute fraction of administered ZUPIVAC H would have gradually reached the brain due to movement of cerebrospinal fluid and diffusion [16]. Then the drug acting on the brain would have initiated GTCS.

### Circumstantial evidence for and against suspecting heavy bupivacaine (ZUPIVAC H) as the cause of her seizures

Our hospital uses about 400–450 ampoules of heavy bupivacaine per month at present. No similar incident happened at our hospital during the past 23 years to our knowledge. ZUPIVAC H brand arrived at our hospital for the first time, 45 days before this incident. Ampoules of batch No. DP2203 arrived at our hospital eight days before this incident. Another patient in our hospital developed refractory status epilepticus to ZUPIVAC H three days apart, arousing suspicion of something unique to ZUPIVAC H contributing to these events. Moreover, our patient had undergone spinal anesthesia with heavy bupivacaine (without adjuvants) five years before for her first LSCS, without complications. Therefore, we suspect that the impurity in the drug also contributed to her seizures. Nevertheless, there are reports of patients who had undergone uneventful spinal anesthesia, developing neurotoxicity during subsequent spinal anesthesia [15, 17]. Forty five patients in our hospital and more patients in other hospitals have undergone spinal anesthesia with ZUPIVAC H of the same batch without a problem on the same day and within the previous seven days. That fact is against suspecting a factor unique to ZUPIVAC H causing this event. Considering all this, we believe that ZUPIVAC H was the likely reason for her side effects and certain patient factors would have made our patient and the other patient of our hospital susceptible to the side effects of ZUPIVAC H.

### Explanation of the patient’s clinical and autopsy findings

Rapid onset of high fever, marked temperature fluctuations ending in death usually occur in central hyperthermia [18]. We think she had central hyperthermia owing to brain damage due to super-refractory status epilepticus plus possibly due to ZUPIVAC H [19–21]. Heat generated in muscles during seizures would have contributed to her fever initially, but when her temperature was highest (day 4) there were no seizures. High fever notwithstanding antipyretics and antibiotics, decline in CRP level from day 2 to day 4, normal CSF analysis results and negative central venous catheter blood and urine culture results indicate that infection is unlikely to be the cause of her hyperpyrexia. However, her neutrophil count was very high on day1 but gradually dropped. Her high neutrophil counts can be attributed to status epilepticus [20].

Neurocardiogenic pulmonary edema following status epilepticus is well documented [20, 21]. That explains her first day CXR changes. Our patient had a cardiac arrest on day 2 and resuscitated and on day 4 undergone a prolonged (> 1 h) cardiopulmonary resuscitation (CPR) before her death was confirmed. Respiratory tract infections (pneumonia) is the most common infection associated with status epilepticus [20].Those would have contributed to the changes in lungs observed at autopsy [20–22].

Rhabdomyolysis following status epilepticus is common and well known [20, 21]. Some drugs given to her like phenytoin and levetiracetam and pre-existing hypothyroidism may have aggravated rhabdomyolysis [23, 24].

Sudden unexpected cardiac arrest and death can happen in uncontrolled epilepsy [25]. Her cardiac arrest on day 2 may be attributable to status epilepticus [25]. The combination of effects of infused thiopentone and any bupivacaine cardiotoxicity also may have contributed to this. Although the echocardiogram did not depict characteristic left ventricular apical akinesia and ballooning, considering ST elevations in her ECG that appeared after doing the echocardiogram, very high troponin I levels and no blocks in coronaries observed at autopsy we think she may have had Takotsubo cardiomyopathy (stress cardiomyopathy) later. The stress of severe illness, first cardiac arrest and being on three inotropes also would have contributed to this. About 50 Takotsubo cardiomyopathy cases have been reported related to seizure activity, including 15 associated with status epilepticus [26]. Interestingly, there are reports of ischemic ECG changes, elevated troponin I with normal coronary angiography without seizures resulting after spinal anesthesia described as bupivacaine induced cardiac toxicity by reporting authors [27, 28].

Histopathological changes in her kidneys and liver are likely to be due to hypoxemia during prolonged resuscitation after the second cardiac arrest. Liver dysfunctions occur in 25% of patients with rhabdomyolysis by various mechanisms including the released proteases causing liver inflammation [29]. This explains her high liver enzyme levels. Drugs like levetiracetam also might have contributed to kidney injury [30]. Her proteinuria is likely to be due to myoglobinuria.

A CSF analysis depicting chemical meningitis was reported in a past patient who had seizures following spinal anesthesia [31]. Nevertheless, our case and some similar cases did not show CSF evidence of chemical meningitis [1–3, 5].

As explained above, her rhabdomyolysis, central hyperthermia, cardiomyopathy and postmortem changes observed in her lungs, kidneys and liver may be largely due to her super refractory status epilepticus with contributions from other factors. Her status epilepticus was due to ZUPIVAC H. That initiated the train of events leading directly to her death. Hence, the side effect of ZUPIVAC H most probably was her underlying cause of death [32]. However, we do not have adequate information to specifically identify the responsible component(s) of ZUPIVAC H.

### Differential diagnoses we considered at different stages

We initially suspected a high spinal or local anesthetic systemic toxicity (LAST). In a patient with short stature and obesity, a high spinal can occur with a regular dose of heavy bupivacaine. The presence of a clear sensory level with preserved handgrip, the absence of any bloody tap, administering only 12.5 mg of bupivacaine, absence of other symptoms of LAST and no initial cardiac involvement indicated that those two possibilities are unlikely. At the end of the day1 and thereafter an adverse reaction to ZUPIVAC H, amniotic fluid embolism, normotensive eclampsia and previously undiagnosed brain pathology were the differential diagnoses. Amniotic fluid embolism is unlikely as at the onset there was no hypoxia with severe respiratory symptoms or cardiovascular collapse. Myoclonus is not the first sign of amniotic fluid embolism. Laboratory tests did not show significant coagulopathy. Later histopathology of the lungs also did not show evidence of amniotic fluid embolism [33]. Normotensive eclampsia is unlikely because there were no prior proteinuria, excess edema, or prodromal symptoms and onset of super-refractory status epilepticus after the delivery that did not respond to vigorous treatment [34]. Normal NCCT brain and later autopsy findings excluded previously undiagnosed brain pathology. Malignant hyperthermia (in addition to seizures) after giving suxamethonium is another possibility. However, reduction of ETCO_2_ to 34-40mmHg range after admission to the ICU, fever spikes appearing on the following day (after the decrease of ETCO_2_) and peaking on day 4 were against this possibility. Meningoencephalitis (infection) was also suspected as a reason for the high fever. The CSF analysis and autopsy findings excluded that possibility. At the end of both institutional death reviews, the final consensus was that this was an idiosyncratic reaction to ZUPIVAC H. After the extensive literature review by the first author, considering all available information, we now believe that this is most probably a case of direct local on-target type (mechanism-based) neurotoxicity on the CNS. As explained later, this can be categorized as an idiosyncratic reaction to ZUPIVAC H as well.

### Discussion of central nervous system toxicity by bupivacaine and possible mechanisms of toxicity

The International Union of Basic and Clinical Pharmacology (IUPHAR) defines an adverse drug reaction (ADR) as an unwanted or harmful reaction experienced following the administration of a drug or combination of drugs under normal conditions of use and is suspected to be related to the drug [35]. Accordingly, this is an ADR. IUPHAR defines a side-effect as any effect caused by a drug other than the intended therapeutic effect [35]. The cesarean section was performed up to the delivery of the baby under spinal anesthesia induced by this drug alone (therapeutic effect was there). Additionally, there was this fatal side effect [35]. The United States Food and Drug Administration (FDA) use the terms adverse reactions and side effects as synonyms [36]. The IUPHAR defines drug toxicity as adverse effects of a drug that occur because the dose or plasma concentration has risen above the therapeutic range, either unintentionally or intentionally [35]. Accordingly, this is very unlikely to be drug toxicity due to the following reasons. She was given a therapeutic dose that has been in use worldwide for a long time [37]. Spinal anesthesia was working well during the onset of the seizures indicating that a certain percentage of drug molecules were bound to spinal cord receptors. Normally, the peak plasma concentration increase is approximately 0.4 mg/l (0.4 µg/ml) for every 100 mg of bupivacaine injected intrathecally and that peak takes about 50 min to occur [38]. Considering that there was no bloody tap, the maximum plasma concentration that possibly would have been achieved in 10–15 min after injection was very much lower than 2–3 µg/ml toxic threshold [38, 39]. Some authors of past similar case reports considered their cases as LAST [3, 4]. Nevertheless, LAST is a term to be used if plasma concentration exceeds the therapeutic range [4, 39–42]. Anesthesiology trainees in Sri Lanka and elsewhere learn > 2 mg/kg as the toxic dose of bupivacaine unless it accidentally gets injected intravascularly [40, 42]. Our patient was administered a far lower dose. Hence, LAST may not be an appropriate categorization for cases like this. Local neurotoxicity by local anesthetics is mentioned by a few past authors [41, 43]. We think our case and similar cases following spinal anesthesia are more likely to be due to a category of direct local on-target type (mechanism-based) neurotoxicity on the CNS [44]. This category of toxicity appears to be occurring at unexpectedly low plasma and CSF concentrations, happens very rarely, thus, patient factors may also be playing a role. A case of cardiac toxicity (without seizures) following only 1.1 mg/kg of bupivacaine, injected cutaneously, was reported in a l-carnitine deficient patient [45]. This is one example of patient factors increasing the risk of bupivacaine toxicity. An experiment demonstrated that administration of supplemental l-carnitine could reverse this risk in rats [46]. Interestingly, l-carnitine deficiency is associated with seizures, cardiomyopathy, rhabdomyolysis, etc. as well [47, 48]. We could not screen her for l-carnitine deficiency.

Toxic reactions usually occurs when the plasma concentration of total (bound and unbound) bupivacaine rise ≥ 2–3 µg/ml (generally ≥ 4 µg/ml) and when the unbound concentration ≥ 0.1–0.2 µg/ml [41, 50]. Nonetheless, there is a report of an experiment where a similar aged healthy woman was slowly intravenously infused with bupivacaine and developed GTCS at a plasma concentration of ≥ 1.1 µg/ml [49]. Cases like ours may have been liable to develop seizures at even lower thresholds due to unidentified factors peculiar to the patient.

Further investigations into future similar cases, for patient factors like l-carnitine and alpha-1-acid glycoprotein deficiency (bupivacaine mostly binds to this protein) etc. would be helpful to clarify the pathophysiology of such events.

Another possible patient factor was either abnormal function or concentration of cell membrane receptors or both. We give an example. In one study, GIRK:Kir3 potassium channels of cell membranes were inhibited within seconds of bupivacaine application [50]. Other voltage-gated potassium channels are also inhibited by bupivacaine [50]. Those channels inhibition increase membrane excitability, which can result in seizures [50]. In one experiment, mice were genetically modified resulting in a lack of similar GIRK2 receptors [51]. They had spontaneous seizures and were prone to pharmacologically induced seizures as well [51]. Had her brain GIRK:Kir3 receptors been sparse, inhibition of existing ones by bupivacaine would have contributed to her seizures [50]. We do not have facilities for further investigation in that line. The impurity appeared to be structurally related to bupivacaine. Thus, it is reasonable to speculate that the impurity might also have inhibited those receptors, perhaps even stronger than bupivacaine. Further studies are necessary to clarify this matter.

Bupivacaine (regular) used for spinal anesthesia comes as a racemic mixture. Had bupivacaine administered to this patient contained more R-(+)-enantiomer, which is more potent that could have been more toxic at a lower dose [52, 53]. We could not test ZUPIVAC H for enantiomers.

95% of bupivacaine in plasma is protein bound [40]. Bupivacaine binds to alpha-1-acid glycoprotein and to a lesser extent to albumin in plasma and in the CSF and unbound bupivacaine is responsible toxic effects [40, 41]. If this patient’s CSF had very low levels of above the proteins due to genetic factors, the unbound bupivacaine concentration would have been higher. Serum alpha-1-acid glycoprotein levels decrease during pregnancy and serum levels are correlated with CSF levels [52, 53]. This is another patient factor that would have facilitated neurotoxicity. Acidosis decreases protein binding of bupivacaine [50]. Initial acidosis would have raised unbound bupivacaine in her CSF. We could not test her plasma and CSF for those two proteins. Her preserved handgrip power and finding a sensory level at Th 7 indicate a considerable fraction of the drug injected remained attached to the spinal cord at the initiation of myoclonus/seizures (only a fraction has ascended to brain level). We could not find literature on the safe ceiling of CSF bupivacaine concentration. Future studies on safe CSF ceilings for local anesthetics would be worthy. Our patient never regained consciousness. No significant histopathological changes were seen in her brain. Several such past cases had changes in their EEG and/or MRI and took several days for neurological recovery [1–5]. Those indicate that some damage to brain neurons which are difficult detect in routine histopathology and take long time to recover, has happened in our patient and probably in other similar cases too.

Our patient had an extremely rarely occurring (novel) reaction likely to be due to bupivacaine that is very difficult to explain by the dose and known pharmacology of the drug. Considering the IUPHAR description of idiosyncrasy (type B ADRs) this is an idiosyncratic reaction as well [35]. Idiosyncratic liver damage due to bupivacaine has been reported [54]. Therefore, our and similar cases described in references 1–7 and 9 may be categorized as idiosyncrasies to bupivacaine. Nonetheless, Idiosyncratic reactions usually do not occur in a few minutes [55]. Immune reaction is a known reason behind idiosyncrasy.

This patient underwent uneventful spinal anesthesia for her first LSCS with bupivacaine five years ago. One plausible explanation is that the impurity may have played a role in precipitating seizures. Another possibility is that the patient factor(s) that made her susceptible to status epilepticus would have become clinically relevant in the recent past, even though those may be hereditary factors. For example, l-carnitine deficiency onset at age 39 has been reported [56].

We believe our analysis of possible patient factors would be helpful to investigators of future such cases to determine the pathophysiology of such seizures /status epilepticus. That will be helpful to improve the management and outcome of such cases.

2.5 ml of 0.5% bupiavacaine without dextrose is also used for spinal anesthesia without problems. As explained above, in this patient anesthesia worked as expected without a high spinal block. According to test results, the density of the batch No.DP2203 samples was 1.026 g/ml (at 20^0^C). Which is much higher than the density of CSF of pregnant women at term (about 1.00030 g/ml) [57]. Therefore, despite of standard dextrose concentration was not there (as pronounced by NMQAL), only a small fraction of administered bupivacaine would have ascended to her brain level and caused seizures. In one study, CSF bupivacaine concentrations for the same spinal block level differed between patients by sixfold after standardized administration of plain bupivacaine 20 mg [58]. That underscores the importance of patient factors in shaping CSF bupivacaine concentrations.

Our patient’s CSF bupivacaine concentration (especially unbound bupivacaine concentration) immediately after injection would have been on the higher side due to patient factors. In combination of other patient factors and probably with the impurity it would have caused direct neurotoxicity (seizures). Seizures resulted in other complications and ultimately her death.

Another possible reason for status epilepticus in our patient is the presence of epileptogenic impurities in the bupivacaine ampoule. We would like to give two examples. Pipecolic acid is used in bupivacaine synthesis [59]. The increase of pipecolic acid and correlated metabolites levels in the brain results in pyridoxine-dependent epilepsy [60, 61]. Interestingly, pyridoxine-dependent epilepsy can result in super refractory status epilepticus [61]. Considering the possibility that some pipecolic acid remained in ZUPIVAC H used for our patient contributing to her seizures, we requested from TGA-Australia to look for that. They did not have facilities to perform relevant tests.

During the process of manufacturing heavy bupivacaine, due to heat, variety of glucose degradation products like 5-Hydroxymethylfurfural could be formed from dextrose [62]. Some of them have neurotoxic and cytotoxic properties after parenteral administration [62, 63]. TGA-Australia, on our request tested for 5-Hydroxymethylfurfural and related substances and their levels in the sample were within the permitted levels as per British Pharmacopeia (BP). TGA-Australia looked for the toxic impurity 2,6-dimethylaniline specified in the BP and Indian Pharmacopoeia, but did not detect this compound in the sample.

### Lessons to be learned and the way forward

Ours is a rare and extreme case, and only six similar cases are in the literature. Nonetheless, there are more published milder cases of likely central nervous neurotoxicity occurring after spinal anesthesia [15]. Published cases are usually the tip of the iceberg. As mentioned in the background, this single case had a big negative impact on the Sri Lankan healthcare system, Sri Lankan society and the reputation of pharmaceutical industry [9]. All considered, it is crucial to do further studies on the broad subject. Also, it is essential to take actions to prevent recurrences considering existing evidence. We do not know whether the impurity detected in ZUPIVAC H has epileptogenic properties when injected into the CSF. Extraction of the impurity, identification and test injection to laboratory animals CSF may be helpful to determine whether that was responsible for seizures in our patient. Early spinal anesthetic agents have not undergone considerable controlled testing for neurotoxicity according to a review article [64]. Such studies done according to present standards on spinal anesthetic agents used currently, especially bupivacaine, may throw light on the pathophysiology of similar cases and potential treatments. Previously, we discussed possible mechanisms that can result in cases like ours. We hope that will give a good idea to investigators of future cases where to focus their attention. If an international body takes the initiative to introduce clear consensus definitions to common terms like drug toxicity, LAST and direct neurotoxicity by local anesthetics, that would benefit the medical community. Initiation of a discussion among anesthesiology community to categorize CNS toxicity after subarachnoid injection of local anesthetics as a special category of side effects is worthy. The risk of similar incidents is not mentioned in some manufacturers’ literature. There is no manufacturer’s literature leaflet in the five ampoule packs of ZUPIVAC H. We think the availability of manufacturer’s literature and mentioning the remote risk of such side effects in the manufacturer’s literature of heavy bupivacaine are essential. Although we are from a different country, TGA-Australia kindly analyzed our ZUPIVAC H samples generating information that would be valuable to healthcare community worldwide. It is a good example of the usefulness of international cooperation in investigating such cases. One recent study shows that impurities (contamination) are the commonest cause of defective medicines in Sri Lanka [65]. Continuous maintenance of a strict quality control process from raw materials to the end product level and adherence to good manufacturing practice protocols by manufacturers can prevent impurities from contaminating bupivacaine. Enhancement of capabilities and effectiveness of the National Medicines Regulatory Authority (NMRA) of Sri Lanka and fully implementation of quality assurance mechanisms always (even during emergency drug procurements and accepting donations of medicines by the Health Ministry) would be useful to ensure the quality, safety and efficacy of bupivacaine at hospitals [65]. Strengthening post marketing surveillance work on anesthetic drugs by NMRA-Sri Lanka is also worthy. Looking for impurities in heavy bupivacaine in the case of an incident like our case is not in the USP monographs for quality tests. We believe if relevant authorities can consider the inclusion of testing for impurities for quality tests it would be worthy. Administration of 20% fat emulsion to act as an intravenous sink to remove bupivacaine from neurons was one therapy we could have used and that had been used to manage similar cases [4, 40, 42]. 20% fat emulsion was unavailable locally. We suggest to keep at least one bottle of 20% fat emulsion in every operating theater to be used in cases like this. Some readers may think that we could have used propofol to induce and maintain general anesthesia because that contains lipid as well. However, reported cases of seizures due to propofol came to the minds of the team members desperately managing status epilepticus at that time and we used time tested thiopentone (thiopental) [66]. Propofol was used to manage several similar patients who survived [1–5, 14]. Thus, we retrospectively think that its benefits might outweigh the risk of aggravated seizures. However, one case treated with thiopentone also survived [7]. Further studies may clarify this issue. Research to develop an antidote for bupivacaine neurotoxicity with better efficacy would be meritorious. During our literature survey, we noticed that several similar cases (seizures) and several deaths had occurred related to spinal anesthesia by accidental administration of tranexamic acid instead of bupivacaine [67]. We have excluded that possibility in our case. However, we would like to draw the attention of the anesthesiology community to that issue and reiterate the importance of always adhering to standard safety procedures when administering spinal anesthesia to avoid such medication errors.

## Conclusions

Neurotoxicity by bupivacaine (ZUPIVAC H) at spinal cord level would have started myoclonus in this patient. After the drug traveled to the brain, neurotoxicity would have resulted in the super-refractory status epilepticus. Super-refractory status epilepticus would have led to the other complications ending in her death. Patient factors are likely to have made her susceptible to neurotoxicity. There are several such potential factors. The impurity in the drug would also have contributed to her status epilepticus. There are several possible mechanisms that would have led to status epilepticus and her death, which we discussed. Awareness of those mechanisms would be useful to investigators in such future cases. Implementation of our aforementioned suggestions would be helpful to minimize similar future incidents.

### Electronic supplementary material

Below is the link to the electronic supplementary material.

Supplementary Material 1

Supplementary Material 2

## Data Availability

﻿All relevant data is incorporated in to the manuscript.

## Abbreviations

ASA: American Society of Anesthesiologists
CNS: Central nervous system
EL/LSCS: Elective lower segment Cesarean section
IV: Intravenous
GTCS: Generalized tonic clonic seizures
ECG: Electrocardiogram
CXR: Chest X ray
ICU: Intensivecare unit
NG: Nasogastric (tube)
NCCT: Non-contrast CT scan
CSF: Cerebrospinal fluid
EEG: Electroencephalogram
INR: International normalized ratio
MRI: Magnetic resonance imaging
CPR: Cardiopulmonary resuscitation
USP: United States Pharmacopeia
NMQAL: National Medicines Quality Assurance Laboratory
TGA: Therapeutic Goods Administration
CRP: C-reactive protein
IUPHAR: International Union of Basic and Clinical Pharmacology
ADR: Adverse drug reaction
FDA: Food and Drug Administration
LAST: Local anesthetic systemic toxicity
IP: Indian pharmacopoeia
BP: British pharmacopeia
