# Supplementary material for: Super-refractory status epilepticus, rhabdomyolysis, central hyperthermia and cardiomyopathy attributable to spinal anesthesia: a case report and review of literature
Source: BMC Anesthesiol. 2024 Apr 6;24:132. doi: 10.1186/s12871-024-02485-x (PMC10998312; doi:10.1186/s12871-024-02485-x)
Supplement: Supplementary file 1 — Supplementary Material 1 [file 12871_2024_2485_MOESM1_ESM.docx]

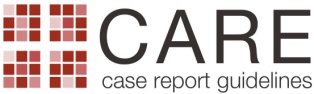
CARE Checklist of information to include when writing a case report
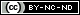


**Topic Item Checklist item description Reported on Line**

**Title 1** The diagnosis or intervention of primary focus followed by the words “case report” 1-2

**Key Words 2** 2 to 5 key words that identify diagnoses or interventions in this case report, including "case report" 46-47

Abstract

**(no references)**

**3a** Introduction: What is unique about this case and what does it add to the scientific literature? 15-18,32-40

**3b** Main symptoms and/or important clinical findings 19-25

**3c** The main diagnoses, therapeutic interventions, and outcomes 32-37

**3d** Conclusion—What is the main “take-away” lesson(s) from this case? 32-40

**Introduction 4** One or two paragraphs summarizing why this case is unique (**may include references**) 50-61

**Patient Information 5a** De-identified patient specific information 64-67

**5b** Primary concerns and symptoms of the patient 72-90

**5c** Medical, family, and psycho-social history including relevant genetic information 64-70

**5d** Relevant past interventions with outcomes 64-70, 309-310

Clinical Findings

**Timeline**

**Diagnostic Assessment**

**Therapeutic Intervention**

**Follow-up and Outcomes**

1. Describe significant physical examination (PE) and important clinical findings 64-116
2. Historical and current information from this episode of care organized as a timeline N/A

**8a** Diagnostic testing (such as PE, laboratory testing, imaging, surveys). 64-126

**8b** Diagnostic challenges (such as access to testing, financial, or cultural) 115-116,135-141

**8c** Diagnosis (including other diagnoses considered) 144-149

**8d** Prognosis (such as staging in oncology) where applicable N/A

**9a** Types of therapeutic intervention (such as pharmacologic, surgical, preventive, self-care) 65-104

**9b** Administration of therapeutic intervention (such as dosage, strength, duration) 69-112

**9c** Changes in therapeutic intervention (with rationale) 69-112

**10a** Clinician and patient-assessed outcomes (if available) 109-126

**10b** Important follow-up diagnostic and other test results 109-126

**10c** Intervention adherence and tolerability (How was this assessed?) 79-126

**10d** Adverse and unanticipated events 76-112

**Discussion 11a** A scientific discussion of the strengths AND limitations associated with this case report 157-348

**11b** Discussion of the relevant medical literature **with references** 150-348

**11c** The scientific rationale for any conclusions (including assessment of possible causes) 157-348

**11d** The primary “take-away” lessons of this case report (without references) in a one paragraph conclusion 144-149

**Patient Perspective 12** The patient should share their perspective in one to two paragraphs on the treatment(s) they received N/A

**Informed Consent 13** Did the patient give informed consent?. Patient died husbands consent was obtained **Yes v No**
