# Supplementary material for: Super-refractory status epilepticus, rhabdomyolysis, central hyperthermia and cardiomyopathy attributable to spinal anesthesia: a case report and review of literature
Source: BMC Anesthesiol. 2024 Apr 6;24:132. doi: 10.1186/s12871-024-02485-x (PMC10998312; doi:10.1186/s12871-024-02485-x)
Supplement: Supplementary file 2 — Supplementary Material 2 [file 12871_2024_2485_MOESM2_ESM.docx]

**Analysis Report: Therapeutic Goods Administration laboratories of Australia.**


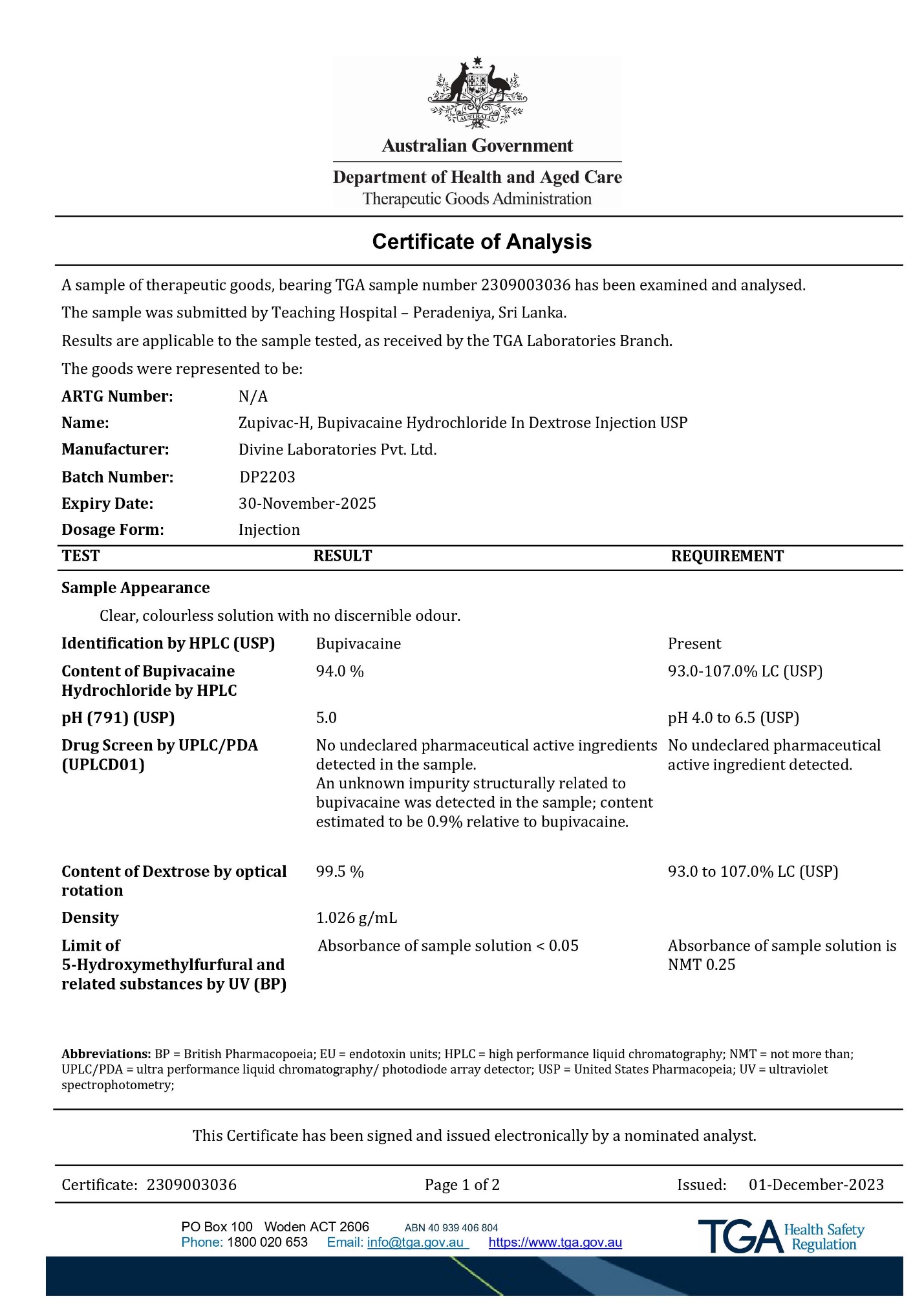


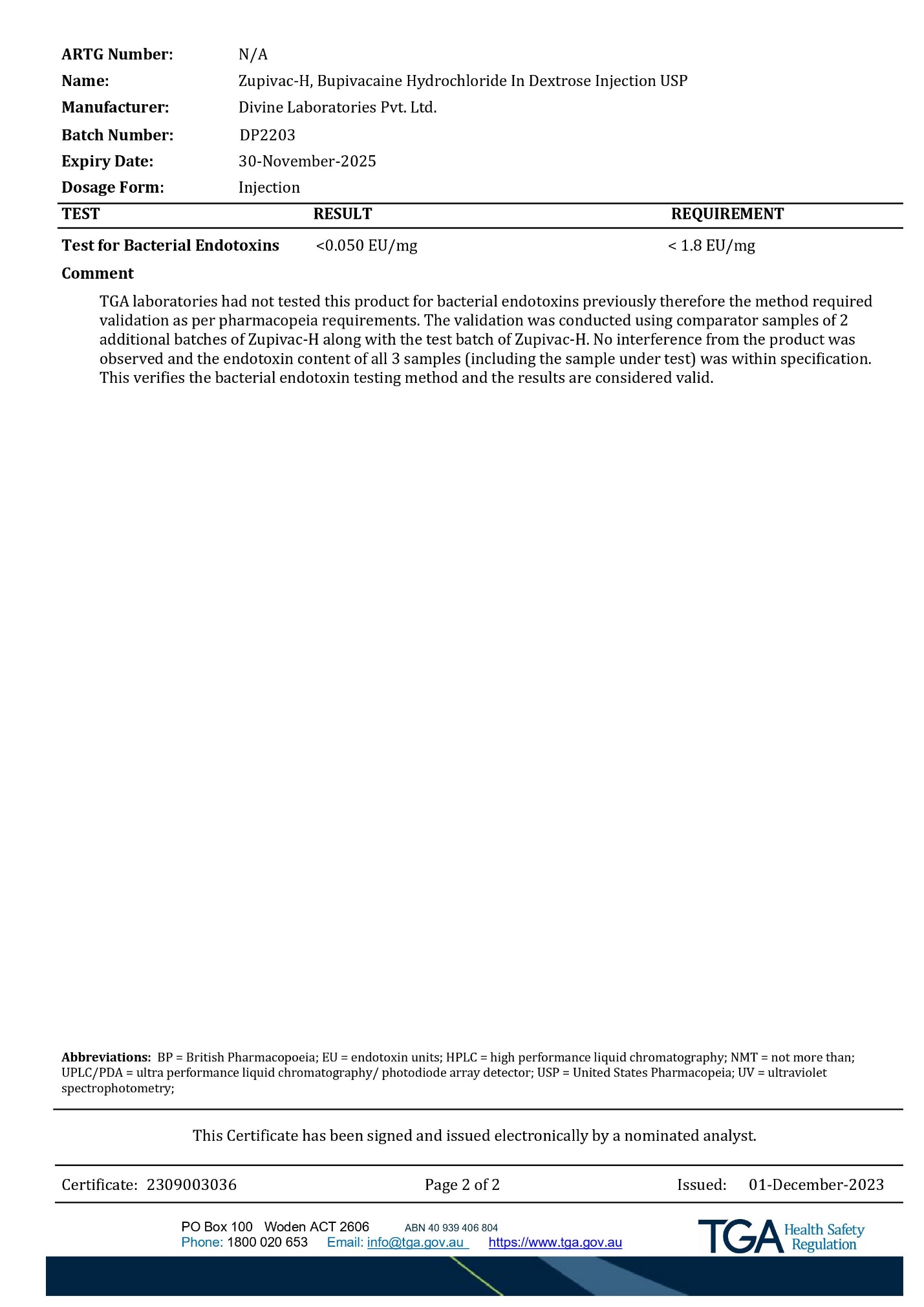


* TGA-Australia looked for the toxic impurity 2,6-dimethylaniline specified in the British pharmacopoeia and Indian pharmacopoeia and did not detect this compound in the sample.

* Impurity which was detected in the batch No. DP 2203: It’s content was estimated to be 0.9% relative to bupivacaine. It’s chemical formula is likely C_15_H_22_N_2_O. TGA has confirmed that this impurity is not mepivacaine. This impurity was not specifically identified in the any of the pharmacopoeias available at TGA-Australia.

* TGA Australia has noticed another unusual thing.The product label claims compliance with the United States pharmacopeia (USP) for the finished product and the Indian pharmacopoeia (IP) for the raw materials which is unusual.
